# An animal study to compare the degree of the suppressive effects on the afferent pathways of micturition between tamsulosin and sildenafil

**DOI:** 10.1186/1423-0127-20-81

**Published:** 2013-10-25

**Authors:** Sung-Eun Kim, Il-Gyu Ko, Lakkyong Hwang, In-Young Choi, Mal-Soon Shin, Chang-Ju Kim, Khae-Hawn Kim

**Affiliations:** 1 Department of Physiology, College of Medicine, Kyung Hee University, #1 Hoigi-dong, Dongdaemoon-gu, Seoul 130-701, Republic of Korea; 2 Department of Urology, Gil Medical Center, Gachon University, #1198 Guwol-dong, Namdong-gu, Incheon 405-760, Republic of Korea

**Keywords:** Overactive bladder syndrome, Tamsulosin, Sildenafil, Neuronal activity, Afferent pathways of micturition

## Abstract

**Background:**

Tamsulosin, an α1-adrenoceptor antagonist, and sildenafil, a phosphodiesterase (PDE) inhibitor, are reported to improve lower urinary tract symptoms including overactive bladder (OAB). This study is aimed at investing the effects of tamsulosin and sildenafil and comparing the degree of the suppressive effects on the afferent pathways of micturition between them using an animal model of OAB, the spontaneously hypertensive rat (SHR).

**Results:**

The cystometric parameters, the basal pressure and duration of bladder contraction, were significantly increased in the SHR group as compared with the Wistar-Kyoto (WKY) group. The intercontraction interval also significantly decreased in the SHR group. In the SHR-Tam 0.01 mg/kg group and the SHR-Sil 1 mg/kg group, however, the basal pressure and duration were significantly reduced and the intercontraction interval was significantly prolonged. Moreover, the degree of the expression of c-Fos and NGF was significantly higher in the SHR group as compared with the WKY group. But it was significantly reduced in the SHR-Tam 0.01 mg/kg group and the SHR-Sil 1 mg/kg group. Furthermore, tamsulosin had a higher degree of effect as compared with sildenafil.

**Conclusions:**

In conclusion, α1-adrenergic receptor antagonists and PDE-5 inhibitors may have an effect in improving the voiding functions through an inhibition of the neuronal activity in the afferent pathways of micturition.

## Background

Overactive bladder (OAB) syndrome is clinically diagnosed based on the presence of urinary urgency [1]. In addition, its symptoms significantly have a negative impact on the emotional well-being and work productivity of affected individuals [2]. Still, however, little is known about its underlying pathophysiology [1].

The bladder and external urethral sphincter are innervated directly or indirectly by the nerve fibers arising from several brain regions including the pontine micturition center (PMC), hypothalamus and preoptic area [3]. The PMC regulates the storage and elimination of urine [4]. The mesencephalic periaqueductal gray matter (PAG) also plays a key role in provoking the micturition reflex, and interneurons in the lumbosacral cord project to the lateral and dorsal parts of the PAG [5]. The central micturition regions, such as the PAG and PMC, are activated by stimulation of the bladder in the OAB group. This increases the expression of c-Fos in the central micturition regions in the brain as compared with normal controls [6].

c-Fos is an immediate early gene, and its expression is triggered by stimuli-induced changes in the metabolic activity of neurons under various conditions [7]. It serves as an indicator for the neuronal activity [6]. It has also been proposed not only that nerve growth factor (NGF) is involved in the neuronal function by which the micturition pathways are modulated but also that its expression serves as an indicator for the bladder overactivity [8].

One of the α1-adrenoceptor blockers, tamsulosin is considered the most effective regimen for patients with lower urinary tract symptoms (LUTS) that are suggestive of benign prostatic hyperplasia [9]. Long-term clinical studies have shown that storage and voiding symptoms were improved when used alone or in combination with other drugs, such as phosphodiesterase-5 (PDE-5) inhibitors [9,10]. Therapeutic potentials of PDE-5 inhibitors including sildenafil for the lower urinary tract dysfunction including OAB have been suggested [11]. According to Ückert and Oelke, randomized, placebo-controlled trials and preliminary open-label studies have addressed the effectiveness of PDE-5 inhibitors in improving the lower urinary tract dysfunction [12]. Thus, these authors reported that it would be a novel approach for the treatment of patients with lower urinary tract dysfunction to modulate the activity of PDE isoenzymes. Of note, sildenafil was effective in improving the LUTS, including detrusor overactivity [13].

Although many studies have reported the efficacies of α1-adrenergic receptor blockers and PDE inhibitors on storage and voiding symptoms, their exact mechanisms of action based on the expression of neuronal activity markers remain unclear [9-13]. Moreover, no studies have compared the degree of the suppressive effects on the afferent pathways of micturition between α1-adrenergic receptor blockers and PDE inhibitors.

In this study, we investigated the effects of tamsulosin and sildenafil and compared the degree of the suppressive effects on the afferent pathways of micturition (the dorsal horn of the L5 spinal cord, ventrolateral periaquaductal gray (vlPAG) and PMC) between them.

## Methods

### Animals and experimental design

We used a total of 32 rats, aged 10 weeks, weighing 250±10 g (24 female SHRs and eight Wistar-Kyoto [WKY] rats) in accordance with the animal care guidelines of the National Institutes of Health (NIH) and the Korean Academy of Medical Sciences (KAMS). We obtained IRB (Institutional Review Board) approval for our study (IRB# 1103–05). We randomly divided our experimental animals into the following four groups:

(1) The WKY group (the control group) (n=8): Water (1 mL) as the vehicle.

(2) The SHR group (n=8): Water (1 mL) as the vehicle.

(3) The SHR-Tam 0.01 mg/kg group (n=8): A 4-week daily course of oral tamsulosin at a dose of 0.01 mg/kg

(4) The SHR-Sil 1 mg/kg group (n=8): A 4-week daily course of oral sildenafil at a dose of 1 mg/kg.

### Blood pressure monitoring and cystometry

Both the SHR and WKY rats were anesthetized with an intraperitoneal injection of Zoletil 50® (10 mg/kg) (Vibac Laboratories, Carros, France) composed of *tiletamine hydrochloride* and *zolazepam hydrochloride*. The rats underwent implantation of a sterile polyethylene catheter (PE50) into the femoral artery, connected to a pressure transducer (Harvard Apparatus, Holliston, MA). Blood pressure monitoring was done using Labscribe (iWork System Inc., Dover, NH).

We performed cystometry as previously described [6]. Here, the cytometric parameters include the intercontraction interval, the basal pressure and duration of bladder contraction, which were measured following an injection of 0.5 mL of saline after the bladder was emptied. Changes in the cystometric parameters were monitored by using Labscribe (iWork System Inc., Dover, NH), and the basal contractions defined as above 5 cmH_2_O were analyzed.

### Histopathological examination and immunohistochemistry

Following the cystometry, the rats were transcardially perfused with 50 mM phosphate-buffered saline (PBS) and then fixed using 4% paraformaldehyde in 100 mM sodium phosphate buffer at pH 7.4. The brain was extracted, postfixed in the same fixative overnight and transferred to a 30% sucrose solution for cryoprotection. This was followed by the preparation of serial coronal sections of 40 μm in thickness using a freezing microtome. The vlPAG and the PMC were selected from the midbrain region spanning from Bregma -7.64 to -8.00 mm and from Bregma -9.68 to -9.80 mm, respectively. Then, the dorsal horn of the L5 spinal cord was selected. In each region, four sections were collected on average from each rat.

To analyze the degree of the expression of c-Fos and NGF in the afferent pathways of micturition, we performed immunohistochemistry as previously described [6]. Coronal sections of brain tissue were incubated overnight with rabbit anti-c-Fos antibody and then treated with mouse anti-NGF antibody (Santa Cruz Biotechnology Inc., Santa Cruz, CA). This was followed by a 1-h incubation with anti-rabbit secondary antibody followed by anti-mouse secondary antibody (Vector Laboratories Inc., Burlingame, CA). Subsequently, the tissue samples were incubated with an avidin-biotin-peroxidase complex (Vector Laboratories) at room temperature for 1 h. For immunohistochemistry, the tissue samples were placed in a solution consisting of 0.02% 3,3’-diaminobenzidine (DAB) tetrahydrochloride (Sigma Chemical Co., St. Louis, MO) and 0.03% H_2_O_2_ in 50 mM Tris–HCl (pH 7.6). Then, the tissue samples were rinsed with PBS three times and mounted onto gelatin-coated slides. The slides were air-dried overnight at room temperature, and the coverslips were mounted by using Permount® (Fisher Scientific, Pittsburgh, PA).

The degree of the expression of c-Fos and NGF in the afferent pathways of micturition was analyzed based on the number of c-Fos- or NGF-positive cells using the Image-Pro® Plus computer-assisted image analysis system (Media Cyberbetics Inc., Silver Spring, MD) installed in a light microscope (Olympus, Tokyo, Japan). We expressed the results as the number of cells/mm^2^ in each region.

### Statistical analysis

Statistical analysis was performed using IBM SPSS (version 20.0; IBM Corp., Armonk, NY). All data are expressed as the mean±SEM (SEM: standard error of the mean). We also performed the one-way ANOVA and Duncan’s *post-hoc* analysis. A *P*-value of <0.05 was considered statistically significant.

## Results

### The cystometric parameters

**Figure 1:**
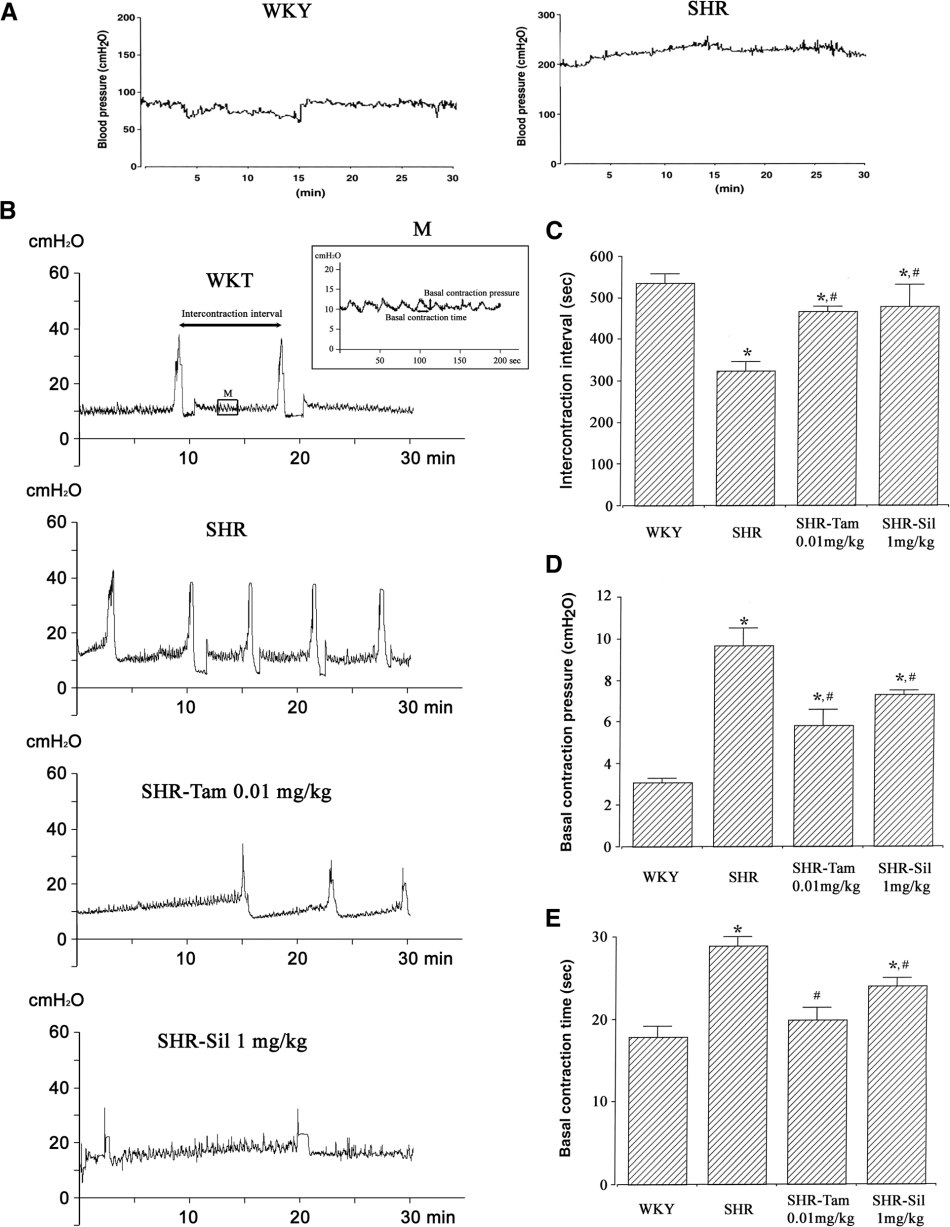
**Changes in the cystometric parameters. (A)** Blood pressure in the Wistar-Kyoto (WKY) rats and in the spontaneous hypertensive rats (SHRs) **(B)** Representative cystometry curves (M represents the magnification of the cystometry curve). **(C)** Changes in the intercontraction interval. **(D)** Changes in the basal contraction pressure. **(E)** Changes in the basal contraction time. *P < 0.05 compared with the WKY group. #*P* < 0.05 compared with the SHR group.

The present results showed that the intercontraction interval (ICI) significantly decreased in the SHR group as compared with WKY group (*P*<0.05). In addition, the basal pressure and duration of bladder contraction were significantly higher and longer in the SHR group (*P*<0.05). But they were significantly reduced in the SHR-Tam 0.01 mg/kg group and the SHR-Sil 1 mg/kg (*P*<0.05). These results indicate that the basal pressure and duration of bladder contraction were significantly reduced following the treatment with tamsulosin or sildenafil. Moreover, Tam 0.01 mg/kg and Sil 1 mg/kg significantly prolonged the ICI (*P*<0.05) (Figure 1).

### The expression of c-Fos and NGF in the dorsal horn of the L5 spinal cord

**Figure 2:**
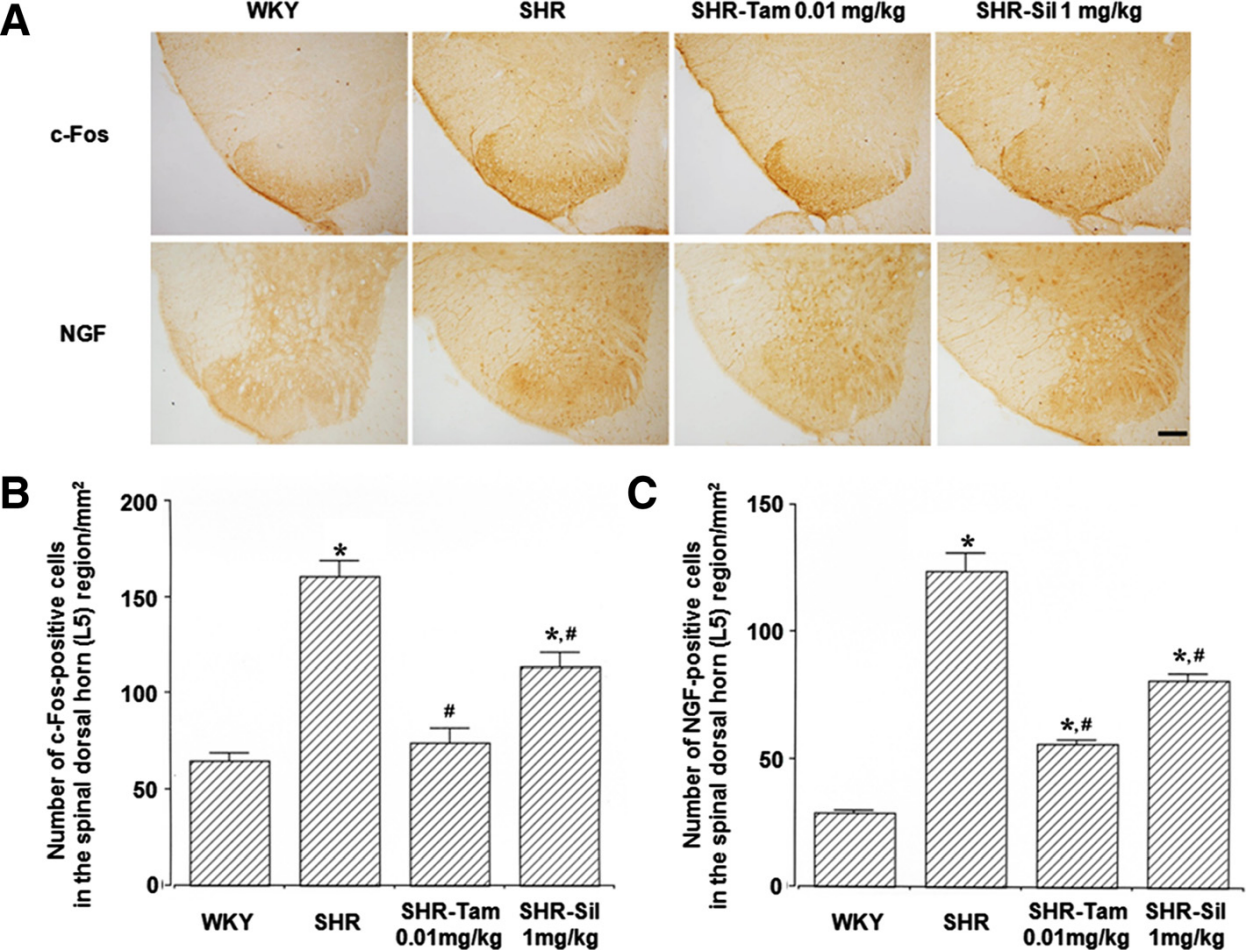
**Changes in the degree of the expression of c-Fos and NGF in the dorsal horn of the L5 spinal cord. (A)** Representative photomicrographs of c-Fos- and NGF-positive cells. The scale bar represents 100 μm. **(B)** Changes in the degree of the expression of c-Fos in the dorsal horn of the L5 spinal cord. **(C)** Changes in the degree of the expression of NGF in the dorsal horn of the L5 spinal cord. *P < 0.05 compared with the WKY group. #*P* < 0.05 compared with the SHR group.

The degree of the expression of c-Fos and NGF was significantly higher in the SHR group as compared with the WKY group (*P*<0.05). But it was significantly reduced in the SHR-Tam 0.01 mg/kg group and the SHR-Sil 1 mg/kg group (*P*<0.05) (Figure 2). These results mean that the enhancement of neuronal activities in the spinal cord may be associated with the bladder overactivity, and Tam 0.01 mg/kg and Sil 1 mg/kg significantly suppressed these activities.

### The expression of c-Fos and NGF in the vlPAG

**Figure 3:**
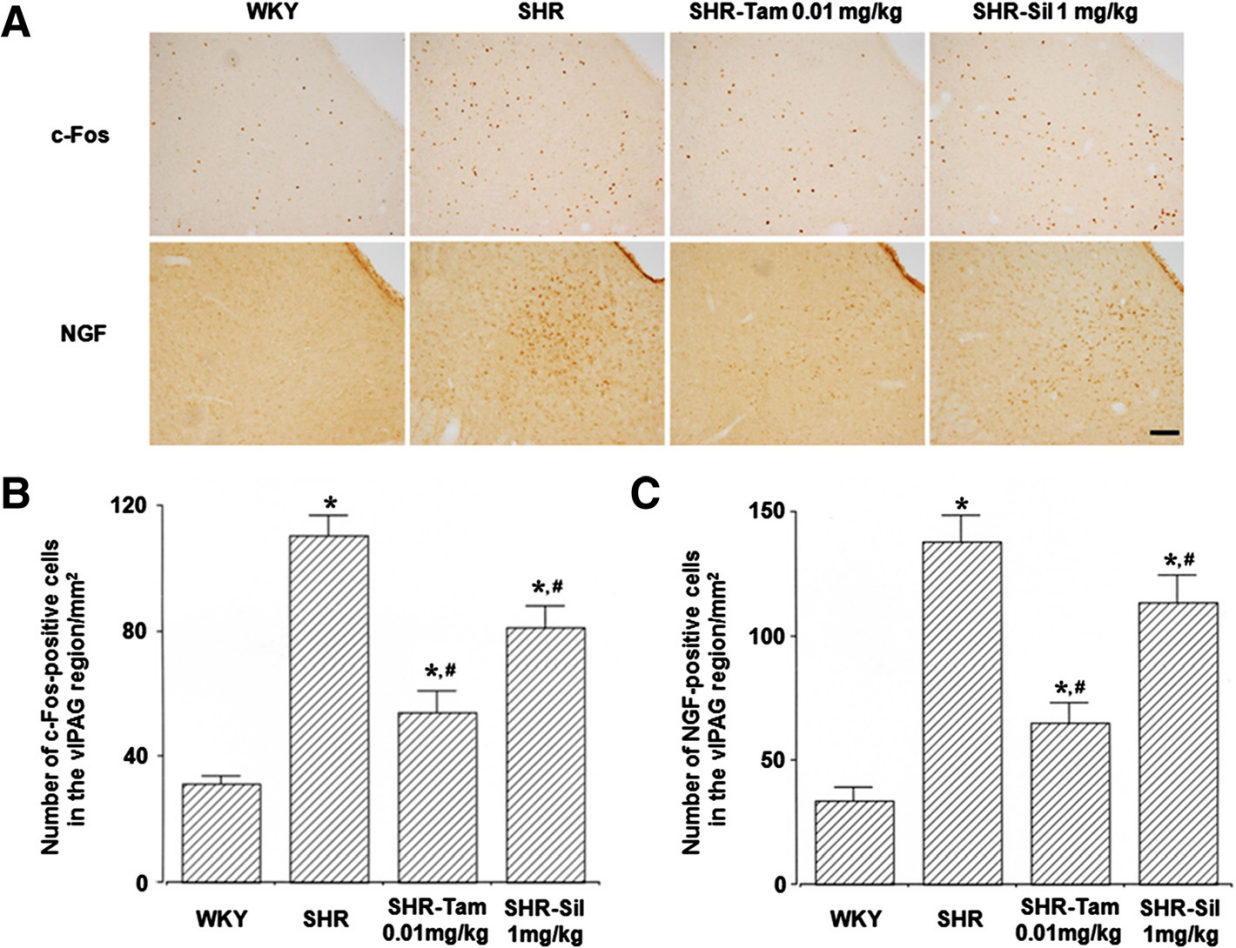
**Changes in the degree of the expression of c-Fos and NGF in the vlPAG. (A)** Representative photomicrographs of c-Fos- and NGF-positive cells. The scale bar represents 100 μm. **(B)** Changes in the degree of the expression of c-Fos in the vlPAG. **(C)** Changes in the degree of the expression of NGF in the vlPAG. *P < 0.05 compared with the WKY group. #*P* < 0.05 compared with the SHR group.

The expression of c-Fos and NGF was significantly enhanced in the SHR group when compared with the WKY group (*P*<0.05). On the other hand, it was significantly reduced in the SHR-Tam 0.01 mg/kg group and the SHR-Sil 1 mg/kg group (*P*<0.05) (Figure 3). These results indicate that the increase in neuronal activities of the vlPAG, a critical component of the micturition reflex, may be involved in the bladder overactivity, and Tam 0.01 mg/kg and Sil 1 mg/kg significantly suppressed these activities.

### The expression of c-Fos and NGF in the PMC

**Figure 4:**
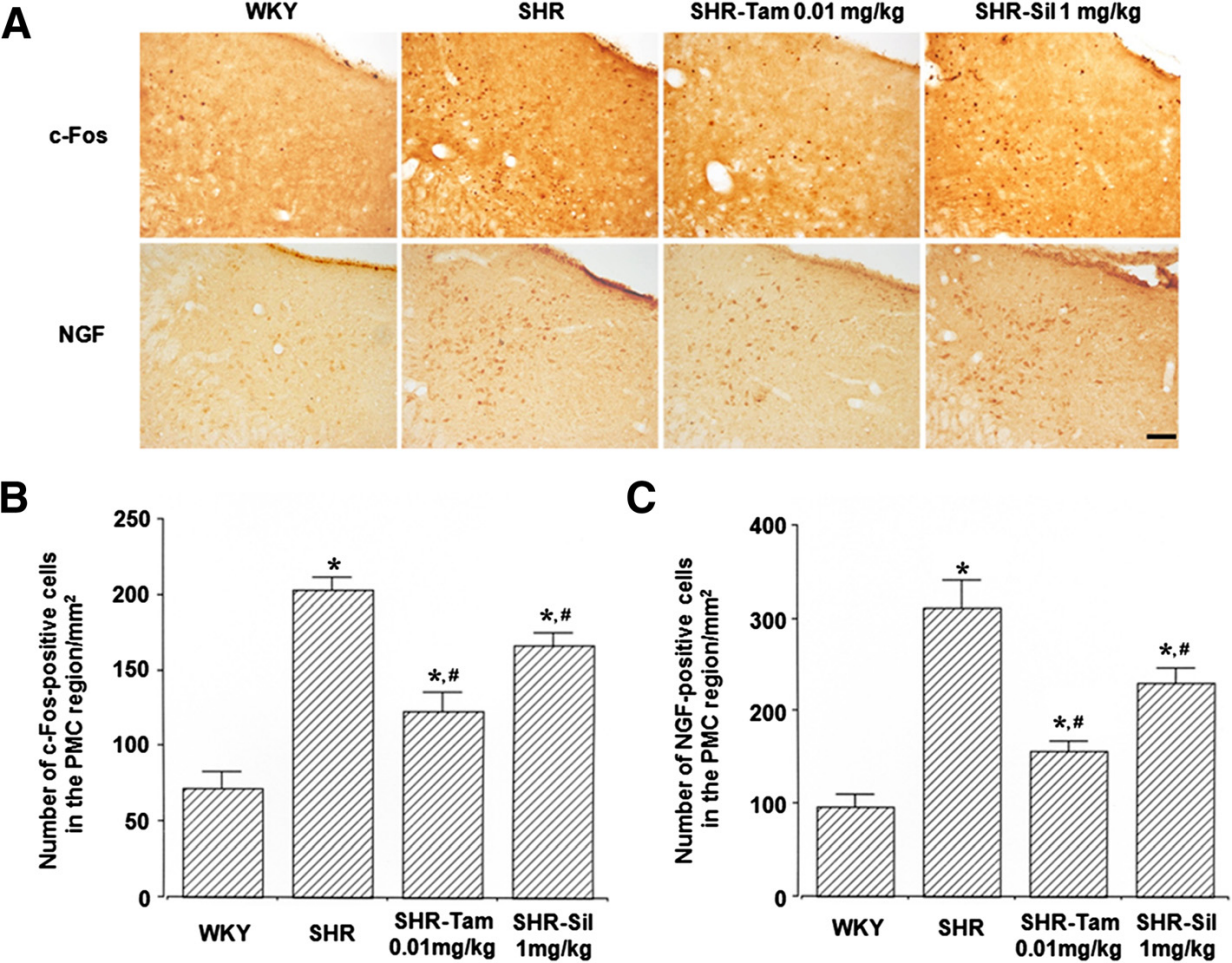
**Changes in the degree of the expression of c-Fos and NGF in the PMC. (A)** Representative photomicrographs of c-Fos- and NGF-positive cells in each group. The scale bar represents 100 μm. **(B)** Changes in the degree of the expression of c-Fos in the PMC. **(C)** Changes in the degree of the expression of NGF in the PMC. *P < 0.05 compared with the WKY group. #*P* < 0.05 compared with the SHR group.

The degree of the expression of c-Fos and NGF was significantly higher in the SHR group as compared with the WKY group (*P*<0.05). But it was significantly reduced in the SHR-Tam 0.01 mg/kg group and the SHR-Sil 1 mg/kg group (*P*<0.05) (Figure 4). From these results, Tam 0.01 mg/kg and Sil 1 mg/kg significantly suppressed the neuronal activities in the central micturirion region, the PMC.

## Discussion

In an animal experimental model of hypertension using SHRs, there were abnormal bladder functions, hyperactive behavior (increased urinary frequency) and the increased occurrence of non-voiding contractions that are suggestive of detrusor overactivity [14]. Presumably, the OAB might originate from the major abnormality of the central nervous system, characterized by alterations in the noradrenergic control of the micturition reflex [15].

According to de Groat and Yoshimura, the expression of c-Fos in the spinal cord is an indicator of the involvement of the spinal neurons in processing afferent signals from the lower urinary tract *via* the spinal reflex pathway [16]. Afferent pathways arising from the lower urinary tract in rats project to the thoracolumbar (T12-L2) and lumbosacral (L5-S1) regions of the spinal cord *via* the hypogastric, pelvic and pudendal nerves [17]. It can therefore be inferred that the increased neuronal activity in the lumbosacral region of the spinal cord might stimulate the micturition centers in the brain. It is noteworthy that the lateral and dorsal parts of the PAG receive the afferent signals from the lumbosacral region of the spinal cord [5]. Then, the afferent signals from the urinary bladder are transmitted to the PAG *via* the neurons in the lumbosacral region of the spinal cord when the bladder is filled with urine. This is followed by the activation of the cells sending a projection to the PMC of the PAG, followed by the micturition [4,18]. In cases of OAB due to the middle cerebral artery (MCA) occlusion, there is an increase in the degree of the expression of c-Fos mRNA in the pontine tegmental area [19]. The pontine tegmentum, also known as the PMC, acts as a switch in the micturition reflex pathway and it thereby controls the bladder capacity and the pressure of bladder contraction [4,20]. With the stimulation of the PMC by excitatory neurotransmitters, bladder contraction is induced and its amplitude is increased. In addition, the threshold bladder volume is reduced [20]. Based on these reports, it can be inferred that OAB symptoms might occur with the stimulation or enhancement of neuronal activity in the PMC and PAG.

NGF modulates the neuronal function *via* the micturition reflex pathway, and it plays a vital role in the pathogenesis of bladder overactivity at the spinal level [21]. Its level is elevated in the bladder, urethral tissue and urine collected from patients with lower urinary tract symptoms (LUTS) including OAB [8,22]. The OAB and hyperexcitability of bladder afferent neurons are greatly dependent on an NGF-induced decrease in A-type K^+^ current density *as well as* elevated NGF levels in the bladder afferent neurons [23]. Our results showed that the degree of NGF expression in the dorsal horn of the L5 spinal cord, vlPAG and PMC was significantly higher in the SHRs as compared with the WKY rats. Taken together, it can be inferred that the enhancement of NGF expression in the afferent pathways of micturition might be induced by the OAB symptom. Similarly to the degree of NGF expression in association with the OAB, the degree of NGF expression in the neuronal voiding centers (PMC and vlPAG) and the dorsal horn of the L5 spinal cord was significantly increased in an animal experimental model of stress urinary incontinence [24].

With the activation of the α1-adrenergic receptor in the bladder, the OAB symptoms are presented. It can therefore be inferred that α1-adrenergic receptor antagonists might be effective in improving the micturition functions [25]. It has been reported that α1-adrenergic receptor antagonists, including prazosin and tamsulosin, were effective in significantly increasing the bladder capacity and lowering the voiding frequency [26]. Moreover, α1-adrenergic receptor antagonists act on the bladder wall and spinal cord and thereby improve the bladder obstruction and the voiding function [25]. But these reports failed to clarify the exact mechanisms by which α1-adrenergic receptor antagonists improve the bladder capacity and voiding functions. According to Haga et al., the degree of c-Fos expression in the spinal cord was significantly higher in the SHRs as compared with the WKY rats [27]. These authors also noted that prazosin had a significant effect in lowering the degree of c-Fos expression in the spinal cord, thus suggesting that α1-adrenergic receptor antagonist inhibits the afferent signals from the lower urinary tract [27]. It has been reported that α1-adrenergic receptor improved the bladder storage function by suppressing the sensory C-fiber afferent limb of the micturition reflex pathway [28].

It is well known that PDE-5 inhibitors improve the LUTS, whose effects might be maximized during bladder filling [14,29,30]. Still, however, little is known about the underlying mechanisms by which the PDE-5 inhibitors have a treatment effect on LUTS or OAB. In addition, no studies clarified their target sites [29]. According to Caremel et al., the PDE-5 inhibitors inhibited the bladder afferent neurons, thus possibly having an effect in improving the LUTS [30]. These authors reported that the nitric oxide (NO)/cyclic guanosine monophosphate (cGMP) signaling pathway is involved in the regulation of the micturition reflex through an inhibitory effect on the activity of afferent nerve fibers, thus providing the potential possibility of developing the treatment agents for OAB based on the NO/cGMP pathway modulators.

There is a possibility that α1-adrenergic receptor antagonists and PDE inhibitors may improve the LUTS including OAB [9]. Still, however, little is known about the underlying mechanisms by which the α1-adrenergic receptor antagonists and PDE inhibitors modulate or affect urinary voiding function in patients with OAB [12].

Based on our results, it can be concluded that α1-adrenergic receptor antagonists and PDE-5 inhibitors may have an effect in improving the voiding functions through an inhibition of the neuronal activity in the dorsal horn of the L5 spinal cord, vlPAG and PMC.

The limitations of the current study are as follows:

(1) We demonstrated that α1-adrenergic receptor antagonist (tamsulosin) and PDE-5 inhibitor (sildenafil) acted on the afferent pathways of micturition and thereby suppressed their effects on the micturition reflex. But we failed to clarify the exact mechanisms that are involved in other micturition regions such as the pelvis and pudendal afferent and efferent nerves, the anterior cingulate gyrus (ACG), medial preoptic nucleus (MPA) and frontal cortex.

(2) We compared the suppressive effects on the neuronal activity between the two drugs in a monotherapy setting. Further animal experimental studies are therefore warranted to compare the suppressive effects on the neuronal activity between α1-adrenergic receptor antagonists and PDE-5 inhibitors when concomitantly used.

(3) We failed to evaluate the dose-effect relationship of sildenafil, which deserves further studies. To clarify the dose-effect relationship, both drugs should be treated at optimal dose. In our preliminary study, we administered tamsulosin and sildenafil at varying doses of 0.01 mg.kg/day, 0.1 mg/kg/day and 1 mg/kg/day and 1 mg/kg/day, 5 mg/kg/day and 10 mg/kg/day, respectively. Thus, we found that the degree of efficacy of tamsulosin and sildenafil was highest when administered at doses of 0.01 mg/kg/day and 1 mg/kg/day, respectively.

## Conclusions

In summary, we demonstrated that α1-adrenergic receptor antagonists and PDE-5 inhibitors may have an effect in improving the voiding functions through an inhibition of the neuronal activity in the afferent pathways of micturition, such as the dorsal horn of the L5 spinal cord, vlPAG and PMC. Moreover, tamsulosin was more effective in alleviating the OAB symptoms by suppressing more neuronal activity in the afferent pathways of micturition as compared with sildenafil. But further prospective clinical studies are warranted to establish their efficacy in patients with OAB.

## Abbreviations

ACG: Anterior cingulate gyrus; cGMP: Cyclic guanosine monophosphate; DAB: 3,3′-diaminobenzidine; L5: 5th of lumbar vertebra; LUTS: Lower urinary tract symptoms; MCA: Middle cerebral artery; MPA: Medial preoptic nucleus; NGF: Nerve growth factor; NO: Nitric oxide; OAB: Overactive bladder; PAG: Periaqueductal gray matter; PBS: Phosphate-buffered saline; PDE: Phosphodiesterase; PDE-5: Phosphodiesterase-5; PMC: Pontine micturition center; SHR: Spontaneously hypertensive rat; Sil: Sildenafil; Tam: Tamsulosin; vlPAG: Ventrolateral periaquaductal gray; WKY: Wistar-kyoto.

## Competing interests

The authors declare that they have no competing interests.

## Authors’ contributions

K-HK conceived and designed this study; S-EK drafted the manuscript and acquired data; C-JK supervised this study and provided the critical revision of the manuscript for important intellectual content; Il-GK and M-SS analyzed and interpreted data; LH and In-YC provided the administrative, technical, or material support. All authors read and approved the final manuscript.
